# Variants of *ACAN* are associated with severity of lumbar disc herniation in patients with chronic low back pain

**DOI:** 10.1371/journal.pone.0181580

**Published:** 2017-07-24

**Authors:** Romain Shanil Perera, Poruwalage Harsha Dissanayake, Upul Senarath, Lalith Sirimevan Wijayaratne, Aranjan Lional Karunanayake, Vajira Harshadeva Weerabaddana Dissanayake

**Affiliations:** 1 Department of Allied Health Sciences, Faculty of Medicine, University of Colombo, Colombo 8, Sri Lanka; 2 Department of Anatomy, Faculty of Medical Sciences, University of Sri Jayewardenepura, Gangodawila, Nugegoda, Sri Lanka; 3 Department of Community Medicine, Faculty of Medicine, University of Colombo, Colombo 8, Sri Lanka; 4 National Hospital of Sri Lanka, Colombo 10, Sri Lanka; 5 Department of Anatomy, Faculty of Medicine, University of Kelaniya, Ragama, Sri Lanka; 6 Human Genetics Unit, Department of Anatomy, Faculty of Medicine, University of Colombo, Colombo 8, Sri Lanka; University of Crete, GREECE

## Abstract

**Introduction:**

Disc herniation is a complex spinal disorder associated with disability and high healthcare cost. Lumbar disc herniation is strongly associated with disc degeneration. Candidate genes of the aggrecan metabolic pathway may associate with the severity of lumbar disc herniation.

**Objectives:**

This study evaluated the association of single nucleotide variants (SNVs) of the candidate genes of the aggrecan metabolic pathway with the severity of lumbar disc herniation in patients with chronic mechanical low back pain. In addition, we assessed the in-silico functional analysis of the significant SNVs and association of their haplotypes with the severity of lumbar disc herniation.

**Methods:**

A descriptive cross sectional study was carried out on 106 patients. Severity of disc herniation and disc degeneration were assessed on T2-weighted mid sagittal lumbar MRI scan. Sixty two exonic SNVs of ten candidate genes of aggrecan metabolic pathway (*ACAN*, *IL1A*, *IL1B*, *IL6*, *MMP3*, *ADAMTS4*, *ADAMTS5*, *TIMP1*, *TIMP2* and *TIMP3*) were genotyped on a Sequenom MassARRAY iPLEX platform. Multivariable linear regression analysis was carried out using PLINK 1.9 software adjusting for age, gender, body mass index and severity of disc degeneration. Four online bioinformatics tools (Provean, SIFT, PolyPhen and Mutation Taster) were used for in-silico functional analysis.

**Results:**

Mean age was 52.42 ± 9.42 years and 69.8% were females. The mean severity of disc herniation was 2.81 ± 1.98. The rs2272023, rs35430524, rs2882676, rs2351491, rs938609, rs3825994, rs1042630, rs698621 and rs3817428 variants and their haplotypes of *ACAN* were associated with the severity of lumbar disc herniation. However, only the rs35430524, rs938609 and rs3817428 variants of *ACAN* were detected as pathogenic by in-silico functional analysis.

**Conclusions:**

SNVs of *ACAN* and their haplotypes are associated with the severity of lumbar disc herniation. Functional genetic studies are necessary to identify the role of these significant SNVs in the pathogenesis of disc herniation.

## Introduction

Lumbar disc herniation is a complex multifactorial spinal condition associated with disability, work time loss and high health related costs [1]. Lumbar disc degeneration is strongly associated with disc herniation [2]. In traditional view, lumbar disc degeneration and herniation are mainly related to age, gender, body mass index (BMI), smoking, physical activity and heavy loading of the spine. Significant associations were identified in our previous study between single nucleotide variants (SNVs) of candidate genes of the aggrecan metabolic pathway and lumbar disc degeneration [3]. Therefore, it is worthwhile to explore the association of SNVs of respective candidate genes (*ACAN*, *IL1A*, *IL1B*, *IL6*, *MMP3*, *ADAMTS4*, *ADAMTS5*, *TIMP1*, *TIMP2* and *TIMP3*) with the severity of disc herniation.

Aggrecan is the main proteoglycan of the intervertebral disc and provides osmotic properties which assist the disc in resisting mechanical compressive loads transmitted along the spine [4]. With aging, the disc undergoes dehydration due to loss of proteoglycan with aging and the degenerative process is further augmented by repetitive damages. This transforms the disc into a degenerative state and probably disc herniation [5]. Interleukins (ILs), matrix metalloproteinases (MMPs), a disintegrin and metalloproteinase with thrombospondin motifs (ADAMTSs) and tissue inhibitor of metalloproteinases (TIMPs) are important molecules in the aggrecan metabolic pathway [6].

Abnormal genetic variants of the candidate genes in the aggrecan metabolic pathway may alter the structural and functional properties of the aggrecan molecule. Although there are several studies which have identified associations of a Variable Number of Tandem Repeats (VNTR) polymorphism of *ACAN* with lumbar disc degeneration and symptomatic lumbar disc herniation [7], there is a lack of studies which have explored the association of SNVs of *ACAN* with the severity of disc herniation. The “T” allele of the rs1042631 variant of *ACAN* is associated with signal intensity of the disc and increased the frequency of disc herniation among the Finnish population [8]. Although the *MMP1* and *MMP3* are associated with disc herniation [9, 10], relationships of SNVs of the *ADAMTS4*, *ADAMTS5*, *TIMP1*, *TIMP2* and *TIMP3* with the severity of disc herniation need to be explored.

Early detection of genetically predisposed individuals for disc herniation might provide opportunity to take necessary precautions in lifestyle related risk factors. Identification of significant SNVs/haplotypes provides probable therapeutic targets for gene therapy in the future. Considering the findings of the previous genetic study on lumbar disc degeneration [3] and current evidence available in the literature, we extended our research to investigate the associations of candidate genes of the aggrecan metabolic pathway with the severity of lumbar disc herniation. The main objective of this study was to determine the associations of single nucleotide variants of the candidate genes of the aggrecan metabolic pathway with the severity of disc herniation in patients with chronic mechanical low back pain. In addition, we assessed in-silico functional analysis of significant SNVs of the candidate genes and associations of their haplotypes with the severity of lumbar disc herniation.

## Methods

### Study design, setting and participants

A descriptive cross-sectional study was carried out on patients with chronic mechanical low back pain who attended the rheumatology clinic, National Hospital of Sri Lanka, Colombo from May 2012 to October 2014. 368 consecutive patients with chronic mechanical low back pain who fulfilled the eligibility criteria were assessed with lateral x-rays of lumbar spine after obtaining written informed consent. Male and female patients (20 to 69 years) having pain/muscle tension or stiffness localised below the costal margin and inferior gluteal folds [11], during day time and worsening in the latter part of the day due to movements [12] for at least three months duration [13] were included in the study. Patients with back pain due to inflammatory causes, visceral origin, systemic infections affecting spine, metabolic bone diseases, fractures in the vertebral column, past surgeries in the spine, and spinal tumours were excluded. 120 patients were selected to undergo MRI scan of lumbar spine and genotyping based on the severity of disc related degenerative changes (disc space narrowing and anterior osteophyte) in the x-ray of lumbar spine [3, 14]. The study was carried out in accordance with the Declaration of Helsinki and with the approval of the Ethics Review Committee of the Faculty of Medicine, University of Colombo.

### Clinical evaluation

An interviewer administered questionnaire was used to record age, gender and clinical examination was used to record body mass index (BMI). Height (cm) and weight (kg) of the patients were assessed with light clothing and without shoes to the nearest 0.1 cm and 0.1 kg, respectively, and BMI was calculated (kg/m^2^) [15]. International cut off values were used for categorisation of BMI (normal, overweight and obese) [16].

### X-ray assessment

**Fig 1:**
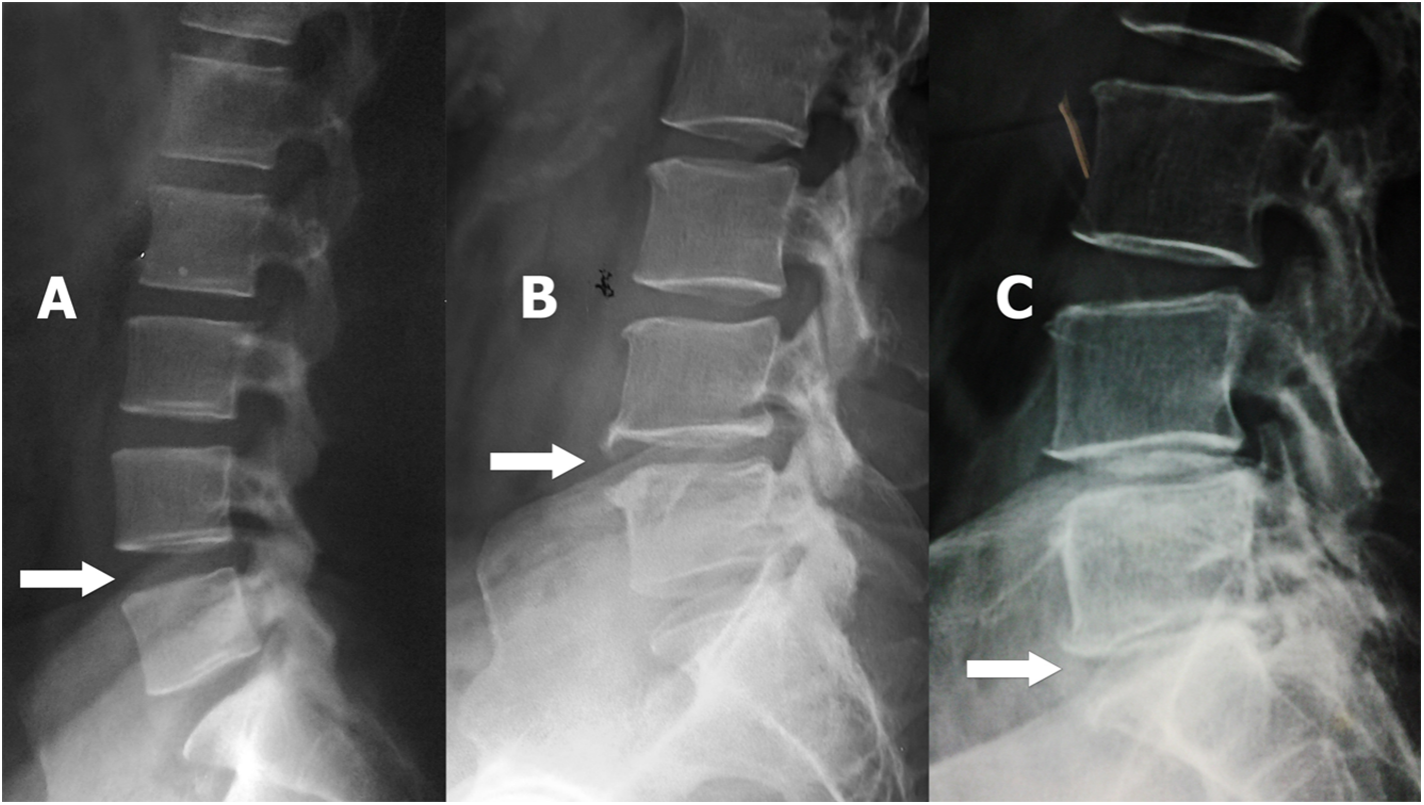
Assessment of the x-ray features of lumbar disc degeneration—lateral x-ray of lumbar spine. Arrows—A–no disc space narrowing/anterior osteophyte (grade 0 lumbar disc degeneration), B–mild disc space narrowing and small anterior osteophyte (grade 1 lumbar disc degeneration), C–small anterior osteophyte and moderate disc space narrowing (grade 2 lumbar disc degeneration) [3].

Lateral lumbar x-rays were carried out while patients were in lateral recumbent position on the table flexing the knees and hips just enough to achieve comfortable position [17]. The intervertebral disc spaces (L1/L2 to L5/S1) in lateral lumbar x-rays were assessed for disc space narrowing and anterior osteophytes by a consultant radiologist blinded to clinical details of the patients and overall lumbar disc degeneration was calculated (grade 0–2) according to a scoring system defined by Lane *et al*. 1993 (Fig 1) [14].

### MRI assessment

Patients with grade 2 or more lumbar disc degeneration in the lateral lumbar x-rays were selected to undergo MRI scan of the lumbar spine. In addition age and gender matched samples from patients with grade 0 and grade 1 lumbar disc degeneration were selected to undergo MRI scans of the lumbar spine. T2 weighted sagittal MRI scans of lumbar spine were carried out in supine position using a GE 1.5T MRI Scanner (Signa, General Electric, Milwaukee, Wisconsin) and assessed for the severity of lumbar disc degeneration and disc herniation.

### Severity of lumbar disc degeneration

The severity of lumbar disc degeneration was graded on T2-weighted mid sagittal MRI images using modified Pfirrmann grading system and each lumbar level was graded from grade 1 to 5 (Table 1) [18, 19]. Grade 2 and 3 was defined as mild lumbar disc degeneration, while grades 4 and 5 were defined as severe lumbar disc degeneration.The grades of lumbar disc degeneration of the five lumbar levels were summed to calculate the severity of lumbar disc degeneration.

**Table 1:** Scoring system to assess the severity of lumbar disc degeneration in T2 weighted midsagittal MRI of lumbar spine.

Grade	Structure	Distinction of nucleus pulposus and annulus fibrosus	Signal Intensity	Height of the disc
1	Homogeneous shape, no horizontal bands	Clear	Hyperintense, isointense	Normal
2	Nonhomogeneous shape with horizontal bands	Some blurring	Hyperintense, isointense	Normal
3	Nonhomogeneous shape	Blurring, but annulus shape still recognizable	Intermediate	Normal to slightly decreased
4	Nonhomogeneous shape	Annulus shape not intact and distinction impossible	Hypointense	Usually decreased
5	Nonhomogeneous shape	Annulus shape not intact and distinction impossible	Hypointense	Collapse disc space

### Severity of lumbar disc herniation

**Fig 2:**
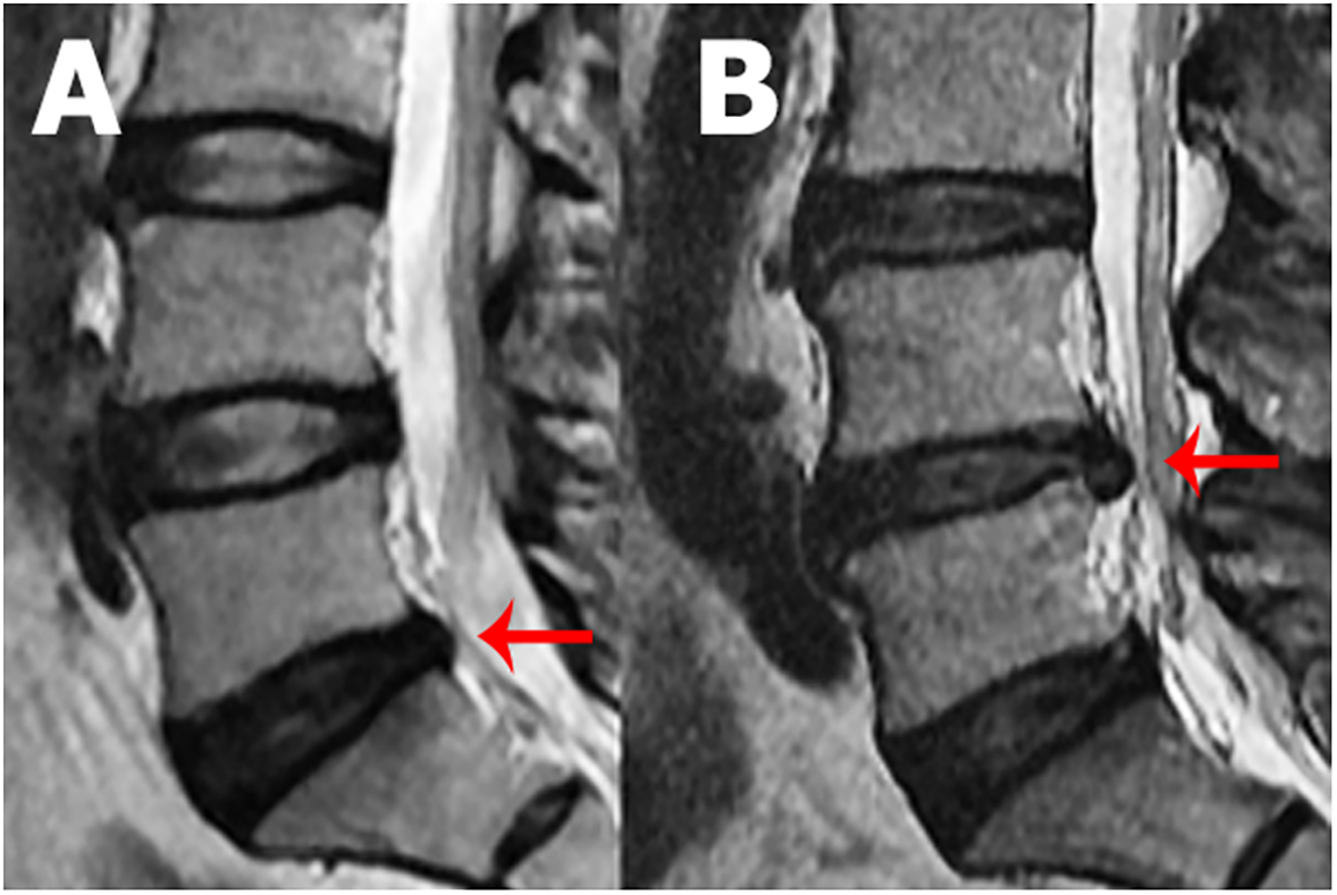
Sagittal MRI of the lumbar spine showing disc protrusion at L5/S1 (A) and disc extrusion at L4/L5 (B).

Disc herniation was considered present when the disc material is displaced beyond the disc space. It was further categorised as disc protrusion when the distance between the edges of the herniated disc material extending outside the disc space is less than the distance between the edges of the base of that disc material. Disc extrusion was considered present when the distance between the edges of the herniated disc material extending outside the disc space was more than the distance between the edges of the base of that disc material (Fig 2) [20]. Presence of disc protrusion in a disc was given a score of 1 and presence of disc extrusion in a disc was given a score of 2. The scores of each lumbar level were summed to calculate the severity of disc herniation.

### SNV selection, DNA extraction and genotyping

The selected molecules of the aggrecan metabolic pathway are aggrecan, interleukin 1α, interleukin 1β, interleukin 6, matrix metalloproteinases 3, a disintegrin and metalloproteinase with thrombospondin motifs 4 and 5, tissue inhibitor of metalloproteinase 1, 2 and 3. Their functions and respective gene symbols are summarised in the Table 1 in the previous article [3]. Common SNVs in the exonic regions of these ten candidate genes in the aggrecan metabolic pathway (*ACAN*, *IL1A*, *IL1B*, *IL6*, *MMP3*, *ADAMTS4*, *ADAMTS5*, *TIMP1*, *TIMP2* and *TIMP3*) were identified from 25 Sri Lankan exomes and Gujarati Indians in Houston (GIH) in the Hapmap database [3]. Sixty two SNVs in exonic regions of the candidate genes in aggrecan metabolic pathway were selected for genotyping (Table 2). DNA was extracted from 200 ml of venous blood using QIAamp DNA Mini Kit in accordance to the blood and body fluid protocol (spin protocol) [21] http://dx.doi.org/10.17504/protocols.io.ihfcb3n. Extracted DNA was quantified using the Quantus fluorometer (Promega) with QuantiFluor® Double stranded DNA system [22] http://dx.doi.org/10.17504/protocols.io.ihgcb3w. Selected variants were genotyped using Sequenom iPLEX MassARRAY system (Sequenom, San Diego, CA) [23] at the Australian Genome Research Facility, Australia. Although there is a VNTR polymorphism of *ACAN* which is associated with the severity of disc herniation, this study focused only on exonic SNVs.

**Table 2:** Selected single nucleotide variants of the candidate genes, their allele frequencies and Hardy–Weinberg equilibrium.

No.	CHR	Position GRCh37/hg19	Gene	Region	SNV	Minor allele	Major allele	MAF %	HWE p value
1	1	161160872	*ADAMTS4*	3’UTR	rs34884997	C	T	0.12	0.68
2	1	161161284	*ADAMTS4*	nonsynonymous	rs41270041	C	G	0.18	0.52
3	1	161163037	*ADAMTS4*	nonsynonymous	rs4233367	T	C	0.18	0.76
4	1	161168004	*ADAMTS4*	synonymous	rs33941127	T	C	0.40	0.85
5	1	161168189	*ADAMTS4*	nonsynonymous	rs34448954	T	C	0.13	0.42
6	2	113532083	*IL1A*	3’UTR	rs2856836	G	A	0.27	0.06
7	2	113532236	*IL1A*	3’UTR	rs1304037	C	T	0.28	0.06
8	2	113537223	*IL1A*	nonsynonymous	rs17561	A	C	0.27	0.06
9	2	113542960	*IL1A*	5’UTR	rs1800587	A	G	0.27	0.06
10	2	113587121	*IL1B*	downstream	rs2853550	A	G	0.29	0.65
11	2	113590390	*IL1B*	synonymous	rs1143634	A	G	0.13	0.69
12	7	22766246	*IL6*	5upstream	rs1800796	C	G	0.46	0.59
13	7	22766645	*IL6*	5upstream	rs1800795	C	G	0.13	0.10
14	7	22771156	*IL6*	synonymous	rs2069849	T	C	0.05	1.00
15	11	102709425	*MMP3*	synonymous	rs520540	A	G	0.33	0.10
16	11	102713465	*MMP3*	synonymous	rs602128	A	G	0.33	0.10
17	11	102713620	*MMP3*	nonsynonymous	rs679620	T	C	0.33	0.21
18	15	89382027	*ACAN*	synonymous	rs372041880	C	A	0.07	0.10
19	15	89382129	*ACAN*	nonsynonymous	rs16942318	A	C	0.05	0.05
20	15	89386652	*ACAN*	nonsynonymous	rs34949187	A	G	0.11	1.00
21	15	89388894	*ACAN*	nonsynonymous	rs148070768	G	A	0.14	0.26
22	15	89388905	*ACAN*	synonymous	rs16942341	T	C	0.07	1.00
23	15	89391160	*ACAN*	synonymous	rs2272023	C	A	0.34	0.42
24	15	89392689	*ACAN*	nonsynonymous	rs144501729	A	C	0.14	0.26
25	15	89398105	*ACAN*	synonymous	rs2351491	T	C	0.37	0.84
26	15	89398553	*ACAN*	nonsynonymous	rs35430524	A	C	0.05	0.05
27	15	89398553	*ACAN*	nonsynonymous	rs3743399	G	A	0.28	0.65
28	15	89398631	*ACAN*	nonsynonymous	rs938609	A	T	0.37	0.70
29	15	89400339	*ACAN*	nonsynonymous	rs2882676	A	C	0.47	0.46
30	15	89400680	*ACAN*	nonsynonymous	rs28407189	G	A	0.07	1.00
31	15	89400963	*ACAN*	nonsynonymous	rs79925540	T	G	0.16	0.48
32	15	89401109	*ACAN*	nonsynonymous	rs4932439	A	G	0.28	0.65
33	15	89401615	*ACAN*	synonymous	rs3825994	G	T	0.44	0.85
34	15	89401616	*ACAN*	nonsynonymous	rs76282091	C	G	0.16	0.48
35	15	89402051	*ACAN*	nonsynonymous	rs1042630	A	G	0.41	0.45
36	15	89402239	*ACAN*	synonymous	rs1042631	T	C	0.35	1.00
37	15	89402596	*ACAN*	synonymous	rs698621	G	T	0.38	0.85
38	15	89415247	*ACAN*	nonsynonymous	rs3817428	G	C	0.05	1.00
39	15	89417238	*ACAN*	nonsynonymous	rs1126823	G	A	0.36	0.84
40	17	76867017	*TIMP2*	synonymous	rs2277698	T	C	0.24	0.80
41	21	28291455	*ADAMTS5*	3’UTR	rs1444269	G	A	0.26	1.00
42	21	28291846	*ADAMTS5*	3’UTR	rs2298657	C	T	0.05	1.00
43	21	28292581	*ADAMTS5*	3’UTR	rs3746836	A	G	0.25	0.81
44	21	28293095	*ADAMTS5*	3’UTR	rs229072	T	A	0.48	0.27
45	21	28293117	*ADAMTS5*	3’UTR	rs229073	G	A	0.48	0.27
46	21	28293924	*ADAMTS5*	3’UTR	rs11700721	T	C	0.12	1.00
47	21	28294090	*ADAMTS5*	3’UTR	rs16979423	G	T	0.14	0.70
48	21	28294143	*ADAMTS5*	3’UTR	rs9978597	G	T	0.04	1.00
49	21	28296135	*ADAMTS5*	3’UTR	rs229078	T	G	0.22	1.00
50	21	28296324	*ADAMTS5*	3’UTR	rs151065	A	G	0.19	0.56
51	21	28296389	*ADAMTS5*	synonymous	rs3746839	G	A	0.08	1.00
52	21	28302355	*ADAMTS5*	nonsynonymous	rs226794	A	G	0.10	0.60
53	21	28338298	*ADAMTS5*	nonsynonymous	rs457947	G	C	0.07	0.46
54	21	28338423	*ADAMTS5*	synonymous	rs55933916	G	C	0.08	0.50
55	22	33253280	*TIMP3*	synonymous	rs9862	T	C	0.50	0.46
56	22	33253292	*TIMP3*	synonymous	rs11547635	T	C	0.07	0.42
57	22	33257322	*TIMP3*	3’UTR	rs1427384	C	T	0.19	0.53
58	22	33258050	*TIMP3*	3’UTR	rs2267184	T	C	0.16	0.48
59	22	33258288	*TIMP3*	3’UTR	rs1065314	C	T	0.17	0.32
60	23	47444879	*TIMP1*	nonsynonymous	rs5953060	C	G	0.43	0.66
61	23	47444985	*TIMP1*	synonymous	rs4898	C	T	0.43	0.66
62	23	47445286	*TIMP1*	nonsynonymous	rs6609533	G	A	0.43	0.66

CHR–chromosome number, SNV—single nucleotide variant, MAF–minor allele frequency, HWE—Hardy Weinberg equilibrium, UTR–untranslated region

### Statistical analysis

Descriptive statistics were used to summarise the sample characteristics. Chi-square test was used to assess the association between two categorical variables while Pearson’s correlation test was used to assess association between two continuous variables. SNVs with Hardy–Weinberg equilibrium (HWE) ≥ 0.05 were included in the genetic association analysis. Quantitative trait association of SNVs was carried out with the multivariable linear regression analysis using PLINK 1.9 software [24]. Severity of disc herniation was used as the quantitative outcome. The genotype of the respective SNV was used as the main independent variable and was treated as a quantitative variable and coded 0, 1 or 2 to represent the number of variant allele, consistent with an additive genetic model. Haplotype frequencies of significant SNVs were performed using PLINK 1.07 software [24], which uses the expectation maximization algorithm to determine common haplotypes. Multivariable linear regression model was used to assess the quantitative trait association of haplotypes of significant SNVs of the candidate genes with the severity of disc herniation. Age, gender, BMI and highest grade of lumbar disc degeneration out of five lumbar levels were used as the additional covariates. Permutation function in PLINK was used with 10,000 permutations to generate the significance levels empirically. It helps to relax the assumptions of normality and correct the errors due to small sample size. P value < 0.05 was used as the level of significance. Four free online bioinformatics prediction tools (Provean [25], SIFT [26], PolyPhen [27] and Mutation taster [28]) were used to conduct the in-silico functional analysis.

## Results

Although 120 patients were selected to undergo MRI scans of lumbar spine, only 106 patients attended for MRI scans of lumbar spine. Characteristics of the patients who underwent both the MRI scan of lumbar spine and genotyping (106 patients) are summarised in Table 3. The proportion of patients increased up to the 50–59 years age group and declined after age of 60 years. The majority of patients were females. Most of the patients were overweight and obese. Mean severity of disc herniation was 2.81 ± 1.98. The majority of patients had disc herniations with 45 (42.4%) having disc extrusions. Among a total of 530 lumbar levels evaluated, 72 (13.6%) and 69 (13.0%) of the discs had severe lumbar disc degeneration and disc extrusions, respectively. Severe lumbar disc degeneration and disc extrusions were more frequent in lower lumbar levels (65.3% and 66.7%, respectively). Severity of disc herniation was positively correlated with the severity of disc degeneration (correlation coefficient = 0.49, p = <0.001). Furthermore, percentage of disc extrusions were higher in discs with severe disc degeneration (33.3%) compared to the discs with mild disc degeneration (9.8%) (χ^2^ (1) = 30.36, p < 0.001). Overall genotyping efficiency of these patients was 98.68%. Minor allele frequency ranged from 0.05 to 0.50 and all the variants were in HWE (Table 2).

**Table 3:** Summary of the sample characteristics of 106 patients who underwent both MRI scan of lumbar spine and genetic association analysis.

Variable		N (%)
Total patients		106
***Socio demographics***		
Age	Mean	52.42 ± 9.42
	20–29 years	3 (2.8)
	30–39 years	7 (6.6)
	40–49 years	22 (20.8)
	50–59 years	51 (48.1)
	60–69 years	23 (21.7)
Gender	Female	74 (69.8)
	Male	32 (30.2)
Body mass index	Normal (18–24.9 kg/m^2^)	37 (34.9)
	Overweight (25–29.9 kg/m^2^)	42 (39.6)
	Obese (≥ 30 kg/m^2^)	27 (25.5)
***MRI assessment***		
Lumbar disc degeneration	Grade 1	0 (0.0)
(maximum grade)	Grade 2	7 (6.6)
	Grade 3	56 (52.8)
	Grade 4	36 (34.0)
	Grade 5	7 (6.6)
Lumbar disc herniation	Yes	97 (91.5)
	No	9 (8.5)
Lumbar disc extrusion	Yes	45 (42.4)
	No	61 (57.6)

### Associations of SNVs of *ACAN* with severity of lumbar disc herniation

Nine SNVs of *ACAN* were significantly associated with the severity of disc herniation (Table 4). Presence of each additional “C” allele of the rs2272023 variant, “A” allele of the rs35430524 variant, “A” allele of the rs2882676 variant and “A” allele of the rs1042630 variant of *ACAN* were associated with a progressive increase in the severity of disc herniation. Furthermore, the presence of each additional “T” allele of the rs2351491 variant, “A” allele of the rs938609 variant, “G” allele of the rs3825994 variant and “G” allele of the rs698621 variant of *ACAN* were associated with progressive reduction in the severity of disc herniation. SNVs, rs2351491, rs938609 and rs698621 variants were in linkage disequilibrium (R^2^ ≥ 0.93, D’ ≥ 0.98). The rs3817428 variant of *ACAN* did not have homozygous genotype for the minor allele and the presence of “G” allele was associated with reduction in the severity of disc herniation. However, there was no significant association between the SNVs of the other candidate genes and the severity of disc herniation (Table 4).

**Table 4:** Severity of disc herniation tabulated according to genotype and the results of multiple linear regression–additive genetic model.

No.	Gene	SNV	Variable	A1	MAF	A2/A2	A1/A2	A1/A1	Standardised coefficient (β)	Adjusted p value
1	*ADAMTS4*	rs34884997		C	0.12					
			Genotypes			T/T	C/T	C/C		
			N			80	23	2		
			DH mean (SD)			2.69 (2.01)	3.09 (1.88)	3.00 (1.41)	0.08	0.397
2	*ADAMTS4*	rs41270041		C	0.18					
			Genotypes			G/G	C/G	C/C		
			N			69	35	1		
			DH mean (SD)			2.78 (1.92)	2.83 (2.09)	1.00 (0.00)	-0.06	0.502
3	*ADAMTS4*	rs4233367		T	0.18					
			Genotypes			C/C	T/C	T/T		
			N			72	29	4		
			DH mean (SD)			2.81 (1.95)	2.79 (1.99)	2.25 (2.63)	0.03	0.764
4	*ADAMTS4*	rs33941127		T	0.40					
			Genotypes			C/C	T/C	T/T		
			N			39	48	18		
			DH mean (SD)			3.03 (2.18)	2.6 (1.85)	2.72 (1.81)	-0.08	0.377
5	*ADAMTS4*	rs34448954		T	0.13					
			Genotypes			C/C	T/C	T/T		
			N			81	20	3		
			DH mean (SD)			2.72 (1.87)	3.2 (2.46)	2.00 (1.00)	0.03	0.753
6	*IL1A*	rs2856836		G	0.27					
			Genotypes			A/A	G/A	G/G		
			N			58	38	9		
			DH mean (SD)			2.95 (2.06)	2.66 (1.95)	2.22 (1.30)	-0.08	0.379
7	*IL1A*	rs1304037		C	0.28					
			Genotypes			T/T	C/T	C/C		
			N			58	37	10		
			DH mean (SD)			2.95 (2.06)	2.73 (1.92)	2.00 (1.41)	-0.10	0.290
8	*IL1A*	rs17561		A	0.27					
			Genotypes			C/C	A/C	A/A		
			N			58	38	9		
			DH mean (SD)			2.95 (2.06)	2.66 (1.95)	2.22 (1.30)	-0.08	0.379
9	*IL1A*	rs1800587		A	0.27					
			Genotypes			G/G	A/G	A/A		
			N			59	38	9		
			DH mean (SD)			3.00 (2.08)	2.71 (1.90)	2.00 (1.50)	-0.09	0.301
10	*IL1B*	rs2853550		A	0.29					
			Genotypes			G/G	A/G	A/A		
			N			57	38	10		
			DH mean (SD)			2.58 (1.66)	3.08 (2.29)	2.80 (2.30)	0.07	0.475
11	*IL1B*	rs1143634		A	0.13					
			Genotypes			G/G	A/G	A/A		
			N			78	27	1		
			DH mean (SD)			2.95 (2.08)	2.48 (1.67)	1.00 (0.00)	-0.07	0.457
12	*IL6*	rs1800796		C	0.46					
			Genotypes			G/G	C/G	C/C		
			N			30	56	20		
			DH mean (SD)			2.60 (2.11)	2.80 (1.85)	3.15 (2.18)	0.03	0.718
13	*IL6*	rs1800795		C	0.13					
			Genotypes			G/G	C/G	C/C		
			N			76	20	4		
			DH mean (SD)			2.68 (1.91)	3.55 (2.21)	1.00 (0.82)	-0.04	0.708
14	*IL6*	rs2069849		T	0.05					
			Genotypes			C/C	T/C	T/T		
			N			94	11	0		
			DH mean (SD)			2.74 (2.02)	3.09 (1.51)	NA	0.06	0.500
15	*MMP3*	rs520540		A	0.33					
			Genotypes			G/G	A/G	A/A		
			N			51	38	16		
			DH mean (SD)			2.88 (2.02)	2.74 (1.96)	2.56 (1.93)	-0.05	0.618
16	*MMP3*	rs602128		A	0.33					
			Genotypes			G/G	A/G	A/A		
			N			51	38	16		
			DH mean (SD)			2.88 (2.02)	2.74 (1.96)	2.56 (1.93)	-0.05	0.618
17	*MMP3*	rs679620		T	0.33					
			Genotypes			C/C	T/C	T/T		
			N			51	39	15		
			DH mean (SD)			2.88 (2.02)	2.72 (1.93)	2.60 (1.99)	-0.05	0.565
18	*ACAN*	rs372041880		C	0.07					
			Genotypes			A/A	C/A	C/C		
			N			92	11	2		
			DH mean (SD)			2.73 (1.99)	3.36 (1.86)	2 (1.41)	0.06	0.498
19	*ACAN*	rs16942318		A	0.05					
			Genotypes			C/C	A/C	A/A		
			N			95	7	2		
			DH mean (SD)			2.85 (2.03)	2.57 (0.79)	1.50 (0.71)	-0.03	0.771
20	*ACAN*	rs34949187		A	0.11					
			Genotypes			G/G	A/G	A/A		
			N			84	20	1		
			DH mean (SD)			2.87 (2.04)	2.45 (1.70)	2.00 (0.00)	-0.13	0.173
21	*ACAN*	rs148070768		G	0.14					
			Genotypes			A/A	G/A	G/G		
			N			75	25	4		
			DH mean (SD)			2.84 (2.01)	2.56 (1.45)	3.75 (3.59)	0.06	0.553
22	*ACAN*	rs16942341		T	0.07					
			Genotypes			C/C	T/C	T/T		
			N			90	15	0		
			DH mean (SD)			2.91 (2.02)	2.00 (1.46)	NA	-0.16	0.084
23	*ACAN*	rs2272023		C	0.34					
			Genotypes			A/A	C/A	C/C		
			N			49	41	15		
			DH mean (SD)			2.67 (1.93)	2.42 (1.86)	4.13 (1.92)	0.22	0.019*
24	*ACAN*	rs144501729		A	0.14					
			Genotypes			C/C	A/C	A/A		
			N			75	25	4		
			DH mean (SD)			2.81 (2.04)	2.56 (1.45)	3.75 (3.59)	0.06	0.546
25	*ACAN*	rs2351491		T	0.37					
			Genotypes			C/C	T/C	T/T		
			N			44	46	15		
			DH mean (SD)			3.39 (2.26)	2.48 (1.67)	1.93 (1.34)	-0.35	<0.001*
26	*ACAN*	rs35430524		A	0.05					
			Genotypes			C/C	A/C	A/A		
			N			96	7	2		
			DH mean (SD)			2.73 (1.92)	2.71 (1.98)	5.50 (3.54)	0.19	0.040*
27	*ACAN*	rs3743399		G	0.28					
			Genotypes			A/A	G/A	G/G		
			N			57	38	10		
			DH mean (SD)			2.84 (2.01)	2.37 (1.84)	4.00 (1.83)	0.13	0.169
28	*ACAN*	rs938609		A	0.37					
			Genotypes			T/T	A/T	A/A		
			N			44	45	15		
			DH mean (SD)			3.37 (2.26)	2.53 (1.65)	1.93 (1.34)	-0.34	<0.001*
29	*ACAN*	rs2882676		A	0.47					
			Genotypes			C/C	A/C	A/A		
			N			32	47	26		
			DH mean (SD)			2.41 (1.78)	2.51 (1.69)	3.73 (2.38)	0.30	0.002*
30	*ACAN*	rs28407189		G	0.07					
			Genotypes			A/A	G/A	G/G		
			N			90	15	0		
			DH mean (SD)			2.91 (2.02)	2.00 (1.46)	NA	-0.16	0.084
31	*ACAN*	rs79925540		T	0.16					
			Genotypes			G/G	T/G	T/T		
			N			74	27	4		
			DH mean (SD)			2.80 (2.05)	2.59 (1.42)	3.75 (3.59)	0.05	0.633
32	*ACAN*	rs4932439		A	0.28					
			Genotypes			G/G	A/G	A/A		
			N			57	38	10		
			DH mean (SD)			2.84 (2.01)	2.37 (1.84)	4.00 (1.83)	0.13	0.169
33	*ACAN*	rs3825994		G	0.44					
			Genotypes			T/T	G/T	G/G		
			N			34	51	20		
			DH mean (SD)			3.32 (2.24)	2.53 (1.72)	2.50 (1.96)	-0.23	0.012*
34	*ACAN*	rs76282091		C	0.16					
			Genotypes			G/G	C/G	C/C		
			N			74	27	4		
			DH mean (SD)			2.80 (2.05)	2.59 (1.42)	3.75 (3.59)	0.05	0.633
35	*ACAN*	rs1042630		A	0.41					
			Genotypes			G/G	A/G	A/A		
			N			40	45	20		
			DH mean (SD)			2.72 (2.02)	2.40 (1.60)	3.75 (2.34)	0.20	0.031*
36	*ACAN*	rs1042631		T	0.35					
			Genotypes			C/C	T/C	T/T		
			N			45	47	14		
			DH mean (SD)			2.93 (2.16)	2.62 (1.75)	3.07 (2.20)	0.05	0.555
37	*ACAN*	rs698621		G	0.38					
			Genotypes			T/T	G/T	G/G		
			N			41	50	14		
			DH mean (SD)			3.24 (2.21)	2.64 (1.82)	1.93 (1.38)	-0.30	0.001*
38	*ACAN*	rs3817428		G	0.05					
			Genotypes			C/C	G/C	G/G		
			N			96	9	0		
			DH mean (SD)			2.94 (1.95)	1.11 (1.27)	NA	-0.23	0.013*
39	*ACAN*	rs1126823		G	0.36					
			Genotypes			A/A	G/A	G/G		
			N			41	51	12		
			DH mean (SD)			2.76 (2.02)	2.88 (1.97)	2.42 (1.98)	0.02	0.860
40	*TIMP2*	rs2277698		T	0.24					
			Genotypes			C/C	T/C	T/T		
			N			60	40	5		
			DH mean (SD)			2.82 (2.08)	2.82 (1.74)	2.00 (2.55)	-0.11	0.266
41	*ADAMTS5*	rs1444269		G	0.26					
			Genotypes			A/A	G/A	G/G		
			N			55	43	6		
			DH mean (SD)			2.82 (2.14)	2.79 (1.74)	2.83 (1.94)	-0.03	0.732
42	*ADAMTS5*	rs2298657		C	0.05					
			Genotypes			T/T	C/T	C/C		
			N			94	9	0		
			DH mean (SD)			2.79 (1.93)	2.44 (2.30)	NA	-0.04	0.671
43	*ADAMTS5*	rs3746836		A	0.25					
			Genotypes			G/G	A/G	A/A		
			N			58	41	6		
			DH mean (SD)			2.74 (2.12)	2.83 (1.77)	2.83 (1.94)	0.01	0.950
44	*ADAMTS5*	rs229072		T	0.48					
			Genotypes			A/A	T/A	T/T		
			N			31	45	27		
			DH mean (SD)			2.58 (2.08)	3.11 (2.01)	2.26 (1.58)	-0.03	0.775
45	*ADAMTS5*	rs229073		G	0.48					
			Genotypes			A/A	G/A	G/G		
			N			31	45	27		
			DH mean (SD)			2.71 (2.16)	3.11 (2.01)	2.26 (1.58)	-0.05	0.613
46	*ADAMTS5*	rs11700721		T	0.12					
			Genotypes			C/C	T/C	T/T		
			N			81	23	1		
			DH mean (SD)			2.69 (2.03)	3.09 (1.78)	3.00 (0.00)	0.04	0.673
47	*ADAMTS5*	rs16979423		G	0.14					
			Genotypes			T/T	G/T	G/G		
			N			78	24	3		
			DH mean (SD)			2.85 (2.02)	2.63 (1.88)	2.33 (1.53)	-0.06	0.544
48	*ADAMTS5*	rs9978597		G	0.05					
			Genotypes			T/T	G/T	G/G		
			N			94	9	0		
			DH mean (SD)			2.80 (1.98)	2.89 (2.09)	NA	0.04	0.657
49	*ADAMTS5*	rs229078		T	0.22					
			Genotypes			G/G	T/G	T/T		
			N			64	35	5		
			DH mean (SD)			2.84 (1.99)	2.94 (19.70)	1.00 (0.71)	-0.03	0.721
50	*ADAMTS5*	rs151065		A	0.19					
			Genotypes			G/G	A/G	A/A		
			N			66	36	3		
			DH mean (SD)			2.81 (1.99)	2.72 (1.89)	2.66 (3.06)	-0.09	0.322
51	*ADAMTS5*	rs3746839		G	0.08					
			Genotypes			A/A	G/A	G/G		
			N			88	16	0		
			DH mean (SD)			2.75 (1.96)	3.12 (1.96)	NA	0.04	0.679
52	*ADAMTS5*	rs226794		A	0.10					
			Genotypes			G/G	A/G	A/A		
			N			84	21	0		
			DH mean (SD)			2.75 (2.04)	2.91 (1.70)	NA	-0.03	0.772
53	*ADAMTS5*	rs457947		G	0.07					
			Genotypes			C/C	G/C	G/G		
			N			92	12	1		
			DH mean (SD)			2.78 (1.99)	2.92 (1.88)	1.00 (0.00)	-0.01	0.890
54	*ADAMTS5*	rs55933916		G	0.08					
			Genotypes			C/C	G/C	G/G		
			N			90	13	1		
			DH mean (SD)			2.77 (2.00)	3.08 (1.85)	1.00 (0.00)	-0.03	0.726
55	*TIMP3*	rs9862		T	0.50					
			Genotypes			C/C	T/C	T/T		
			N			23	58	23		
			DH mean (SD)			2.70 (1.66)	2.76 (1.99)	2.96 (2.27)	-0.03	0.731
56	*TIMP3*	rs11547635		T	0.07					
			Genotypes			C/C	T/C	T/T		
			N			93	11	1		
			DH mean (SD)			2.73 (1.89)	3.09 (2.66)	4.00 (0.00)	0.07	0.464
57	*TIMP3*	rs1427384		C	0.19					
			Genotypes			T/T	C/T	C/C		
			N			67	29	4		
			DH mean (SD)			3.05 (2.11)	2.14 (1.53)	3.25 (1.89)	-0.09	0.358
58	*TIMP3*	rs2267184		T	0.16					
			Genotypes			C/C	T/C	T/T		
			N			76	26	3		
			DH mean (SD)			2.94 (2.07)	2.38 (1.72)	2.33 (0.58)	-0.05	0.601
59	*TIMP3*	rs1065314		C	0.17					
			Genotypes			T/T	C/T	C/C		
			N			74	27	4		
			DH mean (SD)			2.99 (2.06)	2.15 (1.59)	3.25 (1.89)	-0.06	0.548
60	*TIMP1*	rs5953060		C	0.43					
			Genotypes			G/G	C/G	C/C		
			N			43	34	28		
			DH mean (SD)			2.54 (1.75)	2.94 (2.06)	2.96 (2.19)	0.14	0.129
61	*TIMP1*	rs4898		C	0.43					
			Genotypes			T/T	C/T	C/C		
			N			43	34	28		
			DH mean (SD)			2.54 (1.75)	2.94 (2.06)	2.96 (2.19)	0.14	0.129
62	*TIMP1*	rs6609533		G	0.43					
			Genotypes			A/A	G/A	G/G		
			N			43	34	28		
			DH mean (SD)			2.54 (1.75)	2.94 (2.06)	2.96 (2.19)	0.14	0.129

SNV–single nucleotide variant, SD–standard deviation, A1 –minor allele, A2 –major allele, MAF–minor allele frequency, NA–not applicable, β –standardised regression coefficient, data were analysed by multiple linear regression on variant genotypes adjusting for age, gender, body mass index and severity of lumbar disc degeneration

*—p value < 0.05

### In-silico functional analysis of the significant variants associated with the severity of lumbar disc herniation

Among the nine significant SNVs only three (rs35430524, rs938609 and rs3817428) were predicted as pathogenic by the in-silico functional analysis (Table 5). The rs3817428 variant of *ACAN* was identified as pathogenic by two prediction tools (Provean and PolyPhen) while the other two were detected by one of either Provean or PolyPhen prediction tools. Both the rs35430524 and rs3817428 variants were located in conserved regions of *ACAN*. In addition, the rs35430524 variant had a non-conservative amino acid substitution.

**Table 5:** Significant SNVs and their functional predictions.

No	Gene	SNV	Region	AA change	Conservative substitution	Conserved region	Provean	SIFT	Polyphen	Mutant Taster
1	*ACAN*	rs2272023	synonymous			1	Neutral			Harmless
2		rs2351491	synonymous			1	Neutral			Harmless
3		rs35430524	nonsynonymous	P913T	No	0.99	**Deleterious**	Tolerated	Benign	Harmless
4		rs938609	nonsynonymous	S939T	Yes	0.32	Neutral	Tolerated	**Probably damaging**	Harmless
5		rs2882676	nonsynonymous	E1508A	No	0.001	Neutral	Tolerated	Benign	Harmless
6		rs3825994	synonymous			0.19	Neutral			Harmless
7		rs698621	synonymous			0.24	Neutral			Harmless
8		rs1042630	nonsynonymous	I2079V	Yes	0.08	Neutral	Tolerated	Benign	Harmless
9		rs3817428	nonsynonymous	D2373E D2335E	Yes	1	**Deleterious**	Tolerated	**Probably damaging**	Harmless

SNV–single nucleotide variaton; AA–amino acid. Single nucleotide variants with non-conservative substitutions and ones located at conserved regions of a gene have a higher probability to become pathogenic.

### Associations of haplotypes of *ACAN* with the severity of lumbar disc herniation

Among the nine significant SNVs, rs938609 and rs2882676 formed three haplotypes while rs3825994 and rs1042630 also formed three haplotypes. The haplotypes frequencies and results of multivariable linear regression analysis are summarised in Table 6. In haplotype analysis, the T-A haplotype of rs938609 and rs2882676 loci and T-A haplotype of rs3825994 and rs1042630 loci increased the severity of disc herniation. In contrast A-C haplotype of rs938609 and rs2882676 loci and G-G haplotype of rs3825994 and rs1042630 loci reduced the severity of disc herniation after adjusting for confounders (age, gender, BMI and the severity of lumbar disc degeneration).

**Table 6:** Haplotype frequencies and their associations with the severity of lumbar disc herniation.

Haplotypes	Frequency	BETA coefficient	Empirical p value
rs938609-rs2882676			
TA	0.48	0.77	0.001*
AC	0.37	-0.96	<0.001*
TC	0.16	0.15	0.650
rs3825994-rs1042630			
TA	0.40	0.52	0.040*
GG	0.43	-0.66	0.009*
TG	0.16	0.17	0.617

*—p value < 0.05

## Discussion

The study assessed associations of SNVs of candidate genes of the aggrecan metabolic pathway with the severity of lumbar disc herniation in patients with chronic mechanical low back pain. In addition, we assessed the in-silico functional analysis of significant SNVs and associations of their haplotypes with the severity of lumbar disc herniation. In our results, minor allele of the rs2272023 (C), rs35430524 (A), rs2882676 (A), rs2351491 (T), rs938609 (A), rs3825994 (G), rs1042630 (A), rs698621 (G) and rs3817428 (G) variants of *ACAN* were associated with the severity of lumbar disc herniation. Furthermore, the rs35430524, rs938609 and rs3817428 variants were detected as pathogenic by the in-silico functional analysis. In addition, haplotypes of rs938609 and rs2882676 loci, and haplotypes of rs3825994 and rs1042630 loci of *ACAN* were associated with the severity of lumbar disc herniation.

In disc degeneration, the concentration of aggrecan is reduced due to increased breakdown and reduced synthesis of aggrecan molecules leading to disc dehydration [6]. This process is further augmented by concurrent changes of the proteins involved in the aggrecan metabolic pathway (interleukins, matrix metalloproteinases, a disintegrin and metalloproteinase with thrombospondin motifs and tissue inhibitors of metalloproteinases). Annular fissures appear in the disc with the loss of integrity of annulus fibrosus and disc dehydration. Nuclear material can leak through these fissures and displace beyond the disc space margins leading to disc herniation. Disc herniation is categorised as disc protrusion and extrusion based on the extension of herniated disc material beyond the disc space [20]. In our sample, almost all the patients had disc herniations with 42.4% having disc extrusions.

Out of sixty two selected SNVs of ten candidate genes, only nine SNVs of *ACAN* were associated with the severity of disc herniation which included five nonsynonymous variants (rs35430524, rs938609, rs2882676, rs1042630 and rs3817428) and four synonymous variants (rs2272023, rs2351491, rs3825994 and rs698621). A nonsynonymous variant alters the amino acid sequence of the respective protein. This altered amino acid sequence may affect the function of the respective protein. In contrast, synonymous variants do not alter the amino acid sequence of the respective protein, but it can cause changes in long non-coding RNA, microRNA and promoters causing the disease by affecting the protein expression, conformation and function. Furthermore any nonsynonymous or synonymous variant may act as a marker of a functional SNV in the same gene or nearby gene [29]. VNTR polymorphisms reported as significantly associated with the disease are likely to be found as such, because they are in linkage disequilibrium with pathogenic SNVs [30]. Therefore, the focus of this study was to assess the SNVs associated with the severity of disc herniation.

There are a limited number of studies which have explored the association of SNVs of the *ACAN* with the severity of disc herniation. The presence of each “T” allele of the rs1042631 variant of *ACAN* increased the frequency of disc herniation among the Finnish population [8]; it also increased the risk of annular tears in Indian patients who attended a spine unit of a tertiary care hospital [31]. However, this variant was not associated with the severity of disc herniation in our sample. The rs2351491, rs938609 and rs698621 variants of *ACAN* were in strong linkage disequilibrium and each additional minor allele progressively reduced the severity of disc herniation (Table 4). The rs938609 variant is located in exon 12 of *ACAN* and causes a Serine to Threonine substitution at position 939 of the amino acid sequence. This substitution is a very highly conservative substitution, but only the Polyphen bioinformatics tool predicted it as pathogenic (Table 5). The rs2351491 and rs698621 variants are synonymous variants and functionally unremarkable.

The presence of each additional minor allele of the rs35430524 (A), rs1042630 (A) and rs2882676 (A) variants of *ACAN* was associated with a progressive increase in the severity of disc herniation while the presence of an additional “G” allele of the rs3817428 variant of *ACAN* reduced the severity of disc herniation. All these four variants were nonsynonymous, but only the rs35430524 and rs3817428 variants were predicted as pathogenic. The rs35430524 variant is located in exon 12 of *ACAN* and causes a Proline to Threonine substitution at position 913 of the amino acid sequence. It is located at a highly-conserved region of *ACAN* and is not a conservative substitution. Therefore there is a high probability that the rs35430524 variant is pathogenic and which was identified as such by the Provean prediction tool (Table 5). The rs3817428 variant located in exon 15 of *ACAN* causes an Aspartic acid to Glutamic acid substitution at position 2373 of the amino acid sequence. This is located in a highly-conserved area in *ACAN*, but its amino acid substitution is a highly conservative substitution. Two of the four bioinformatics tools (Provean and Polyphen) predicted that the rs3817428 variant is pathogenic (Table 5). The rs3825994 and rs2272023 variants of *ACAN* are synonymous variants and the results of the functional prediction tools were unremarkable.

Our findings on the associations of SNVs of *ACAN* with the severity of lumbar disc herniation were further strengthened by the results of the haplotype analysis. In haplotype analysis, we found that T-A (rs938609—rs2882676) and T-A (rs3825994—rs1042630) haplotypes increased the severity of disc herniation while A-C (rs938609-rs2882676) and G-G (rs3825994- rs1042630) haplotypes reduced the severity of disc herniation. This suggests that the severity of disc herniation is influenced by single nucleotide variation and also by haplotype differences.

Several studies reported significant associations of SNVs of *IL6*, *IL10* [32], *MMP1* and *MMP3* [9] with disc herniation. Some of these variants were intronic variants [32] and some variants were not polymorphic in our population. We have assessed only the exonic variants and the SNVs of the candidate genes of inflammatory, catabolic, anti-catabolic molecules were not associated with the severity of disc herniation (Table 4). In our previous study, SNVs of *IL1A* (inflammatory gene), *ADAMTS4* and *ADAMTS5* (catabolic genes) were found to be associated with the severity of disc degeneration [3], but these SNVs were not associated with the severity of disc herniation. Thus, findings of the two studies suggest that SNVs of candidate genes in inflammatory and catabolic pathways are responsible for the variation in the severity of disc degeneration while SNVs of *ACAN* (structural gene) are responsible for the variation in the severity of disc herniation.

Quantitative traits like disc herniation may have significant effects from variants of several candidate genes and environmental factors. However, we have not corrected the genetic association analysis for the multiple variants we already assessed and other probable variants in other respective genes. Although the rs3817428, rs35430524 and rs938609 variants of *ACAN* were predicted as pathogenic by the in-silico functional analysis, overall results of the four prediction tools were inconsistent. Considering these facts, it is important to carry out genetic association analysis with a larger sample number using whole genome sequencing followed by functional genetic studies to identify the exact effect of the significant SNVs.

### Limitations of the study

The results of this study are not generalizable as it was conducted on a specific group of patients with chronic mechanical low back pain. However, there were patients representing the three districts of the Western Province of Sri Lanka. Low sample number is another limitation of the study, but we used permutation function of the PLINK software tool to relax the assumptions of normality and correct the errors due to small sample size. There may be other confounding factors that we have not corrected for.

## Conclusions

Single nucleotide variants of *ACAN* and their haplotypes are associated with the severity of lumbar disc herniation. The rs35430524, rs938609 and rs3817428 variants of *ACAN* are predicted as pathogenic by in-silico functional analysis. However functional involvement of these three SNVs should be confirmed with functional genetic studies. This study provides basic insight of genetic markers which could be used as probable therapeutic targets for novel treatment strategies for lumbar disc herniation.

## Supporting information

S1 Data Genotype data of 106 patients. (XLSX)
